# Transfer-Entropy- and Hawkes-Process-Driven Dynamic Measurement of Cross-Border Financial Risk Contagion in Directed, Weighted Networks

**DOI:** 10.3390/e28080887

**Published:** 2026-08-06

**Authors:** Lei An, Jinping Dai

**Affiliations:** 1 School of Finance, Nanjing Audit University, Nanjing 211815, China; 2 School of Economics, Nankai University, Tianjin 300071, China; daijp@nankai.edu.cn

**Keywords:** transfer entropy, Hawkes process, directed weighted network, cross-border financial contagion, systemic risk, von Neumann graph entropy, sovereign credit default swap

## Abstract

Quantifying the direction, strength and temporal clustering of cross-border financial risk contagion calls for methods that go beyond linear correlation. We suggest a two-layer framework that brings together transfer entropy and a multivariate Hawkes self-exciting point process on a time-varying, directed, weighted network. In the first layer, one-to-one transfer entropies of sovereign credit default swap spreads are estimated with a bias-corrected *k* nearest neighbour estimator, and this step detects nonlinear and directional information transfer between spreads. The second layer is a multivariate Hawkes process that models how extreme loss events arrive and mutually excite one another across countries, and it gives an excitation intensity matrix, encoding the way a tail event in one country raises the likelihood of an instantaneous hazard occurring in another. By merging these two layers, we obtain a composite, directed, weighted adjacency matrix in which the weights of the edges reflect both information flow and event clustering. We introduce a network-level contagion intensity index and split it into direct, indirect and feedback terms using the graph Laplacian spectrum. Von Neumann graph entropy together with the spectral gap ratio serve as entropy-based measures of the complexity and fragility of the evolving network. We validate the choice of Shannon-type entropy through a Tsallis *q*-sensitivity analysis, and we verify the nonlinear dependence structure of the data using BDS tests and maximal Lyapunov exponent estimates. Three empirical findings emerge from analysing 20 sovereign CDS markets from January 2015 to December 2025: (i) directional risk spillover signals derived based on transfer entropy are more timely than those derived from variance decomposition; (ii) the Hawkes excitation component amplifies measured contagion intensity by 35 to 58 percent during the COVID-19 shock and the 2022 European energy crisis relative to a transfer-entropy-only baseline; (iii) von Neumann graph entropy reaches historically extreme values 7 to 12 trading days before the peak drawdown in a Global Sovereign Bond Index. These results hold across rolling window lengths, significance thresholds, alternative entropy functionals and alternative Hawkes kernels.

## 1. Introduction

The rapid cross-border transmission of adverse shocks is well documented, as was seen during the 2008 global financial crisis, the 2010 European sovereign debt crisis, the COVID-19 pandemic in 2020 and the 2022 European energy crisis. For early warning systems and macroprudential policy design, it is important to understand how contagion is transmitted, how strong it is and how it changes over time. Cross-border financial risk contagion has accordingly become a central concern for policymakers and market participants in an increasingly interconnected global financial system.

Variance decompositions derived from vector autoregressive models represent a classical approach to measuring financial connectedness. The framework of Diebold and Yilmaz [1] decomposes generalised forecast error variances into a spillover matrix and has become a benchmark for directional connectedness measurement; a recent refinement by Diebold and Yilmaz [2] extends the original framework with refined network topology measures. Frequency-domain extensions by Baruník and Křehlík [3] further show that short-run and long-run spillovers can behave quite differently. Demirer et al. [4] scaled the method to a large global bank network, and Bostanci and Yilmaz [5] used it in the context of sovereign credit risk. Engle’s dynamic conditional correlation model [6] remains another widely adopted linear dependence structure; flexible DCC-GARCH specifications for asset allocation were later developed by Billio, Caporin and Gobbo [7], and Gabauer [8] suggested dynamic asymmetric pairwise connectedness measures within an optimal currency area. The regime-switching extensions of DCCs, such as the Markov-switching DCC (MS-DCC) model of Pelletier [9], allow correlation structures to shift abruptly between regimes and thereby detect a form of structural nonlinearity. While informative, these approaches do not fully detect the nonlinear and asymmetric information transfer present in sovereign CDS markets; although GARCH-family volatility specifications embed nonlinearity in conditional variance, the mean equation and the connectedness decomposition remain linear, limiting the ability to identify higher-order and directional dependence.

Transfer entropy, introduced by Schreiber [10], is a model-free, information-theoretic measure of directional dependence. It generalises Granger causality to nonlinear and non-Gaussian settings. Dimpfl and Peter [11] used it in equity markets and demonstrated that it identifies the information flows missed by linear measures. Subsequent work has extended transfer entropy to cryptocurrency markets [12], foreign exchange [13] and financial risk networks [14]. Scagliarini et al. [15] pushed the analysis further to synergistic information transfer across the global system of financial markets. The *k* nearest neighbour estimator of Kraskov, Stögbauer and Grassberger [16] (hereafter, KSG) gives a bias-corrected plug-in estimator with good finite-sample properties, and a thorough treatment of transfer entropy in complex systems is given by Bossomaier et al. [17].

In a separate study, self-exciting point processes were employed to model the clustering of financial stress events. Hawkes processes were introduced by Hawkes [18]; their use for modelling mutual excitation in finance was pioneered by Aït-Sahalia, Cacho-Diaz and Laeven [19]. Bacry, Mastromatteo and Muzy [20] gave a comprehensive overview of Hawkes processes in finance. Branching ratios were used to quantify reflexivity by Filimonov and Sornette [21], and Hardiman and Bouchaud [22] suggested a branching ratio approximation for the self-exciting process. Early multivariate applications to financial data appear in the work of Embrechts, Liniger and Lin [23], and the role of order-book volume was studied by Rambaldi, Bacry and Lillo [24]. More recent extensions include Hawkes-driven limit-order-book modelling [25], sovereign CDS contagion [26] and neural-network-augmented Hawkes processes [27].

Network science has supplied a third pillar. Granger-causality-based connectedness measures for the finance and insurance sectors were introduced by Billio et al. [28], who also employed nonlinear Granger causality tests to detect regime-dependent linkages among financial institutions; the DebtRank measure of systemic importance was suggested by Battiston et al. [29]. Adrian and Brunnermeier [30] developed CoVaR as a conditional tail risk measure, and Brownlees and Engle [31] suggested SRISK as a conditional capital shortfall measure. The theoretical foundations linking network topology to systemic stability were laid by Acemoglu, Ozdaglar and Tahbaz-Salehi [32], and the toolkit has since been enriched by multilayer network analysis [33], temporal extreme risk spillover networks [34] and graph-neural-network-based systemic risk measures [35].

Complementary approaches have brought together tail-event-driven network methods and information-theoretic tools. The TENET framework of Härdle, Wang and Yu [36] uses quantile regression together with network topology to measure tail-event-driven systemic risk. Cross-correlation changes have been suggested as systemic-risk indicators [37], and interconnectedness analyses have been used in the context of Chinese financial institutions [38] and cryptocurrency portfolio management [39]. Xu et al. [40] used DCC-MIDAS models to measure systemic risk in the Chinese banking sector. Markov-switching GARCH–copula models (MS-GARCH–copula) have been developed to jointly detect regime changes in volatility and nonlinear tail dependence across markets [41], and dynamic copula approaches [42] offer a flexible characterisation of joint distribution beyond linear correlation. Entropy-based characterisations of network topologies, including spectral entropies [43] and thermodynamic graph polynomials [44], such as spectral entropies [43] and thermodynamic graph polynomials [44], quantify the information content of the network structure itself. Information entropy has also been linked to market risk measurement [45]. Bouri et al. [46] investigated return connectedness across asset classes around the COVID-19 outbreak and demonstrated that the pandemic substantially restructured global risk transmission channels.

Three gaps nonetheless remain. Earlier transfer entropy work on financial contagion usually constructs undirected or weakly directed information-flow networks and does not account for the temporal self-excitation and mutual excitation that Hawkes models can detect. At the same time, Hawkes-based contagion studies focus on event arrival rates and do not exploit the broader information flow measured by transfer entropy. Furthermore, very little research integrates both tools into a unified network framework equipped with entropy-based complexity indicators that can serve as early warning signals. In addition, existing studies rarely verify that the underlying data exhibit the nonlinear serial dependence that motivates nonlinear methods, nor do they examine the sensitivity of entropy-based indicators to alternative entropy functionals such as Tsallis entropy [47].

By suggesting a two-layer framework that brings together transfer entropy and multivariate Hawkes processes on a time-varying, directed, weighted network, we address these gaps.

On the basis of the above literature, three testable hypotheses guide the empirical analysis.

**Hypothesis** **1:** *A composite network that fuses transfer entropy with Hawkes excitation provides higher measured contagion intensity during stress episodes than either single-layer network alone.*

**Hypothesis** **2:** *Entropy-based structural indicators derived from the composite network give earlier warning signals of systemic stress than the Diebold–Yilmaz total connectedness index.*

**Hypothesis** **3:** *The two-layer contagion measurement is found to be robust when considering the choice of rolling window length, extreme event threshold, Hawkes kernel specification and entropy functional.*

The specific contributions are as follows.

1. We build a rolling-window, bias-corrected transfer entropy adjacency matrix that detects the directional, nonlinear information flow between 20 sovereign CDS markets, complemented by a Hawkes excitation adjacency matrix that estimates the clustering and mutual excitation of extreme tail events (Section 2.2 and Section 2.3).
2. We combine the two layers into a composite, directed, weighted adjacency matrix and decompose the network-level dynamic contagion intensity index into direct, indirect and feedback terms using the graph Laplacian spectrum (Section 2.4).
3. We supply entropy-based indicators to characterise how the contagion network evolves in complexity and fragility over time.
4. A full empirical analysis of 20 sovereign CDS markets from 2015 to 2025 is supplied, and it shows that the two-layer framework outperforms single-layer alternatives in early warning power, contagion intensity measurement and robustness (Section 4). We further validate the nonlinear dependence structure of the data through BDS tests and Lyapunov exponent estimates, and the robustness of entropy-based indicators to the Tsallis *q*-parameter is confirmed.

Table 1 positions the suggested framework against representative prior studies across six dimensions.

In the “Nonlinear Measure” column, ✓ denotes a method that is fully nonlinear in both mean and dependence structure, while Δ indicates partial nonlinearity. The Diebold–Yilmaz framework derives volatilities from GARCH, which introduces nonlinearity in conditional variance, but the connectedness decomposition is based on a linear VAR. Billio et al. [28] supplement linear Granger causality with nonlinear causality tests in later extensions, and Pelletier [9] embeds regime switching in DCC, thereby detecting structural nonlinearity in correlation dynamics.

The remainder of the paper is organised as follows. Section 2 constructs the two-layer framework, covering transfer entropy estimation, Hawkes process modelling and the composite network. Section 3 develops the entropy-based analysis. The empirical results appear in Section 4, policy implications are discussed in Section 5, and Section 6 concludes.

## 2. Two-Layer Framework for Contagion Network Construction

**Figure 1:**
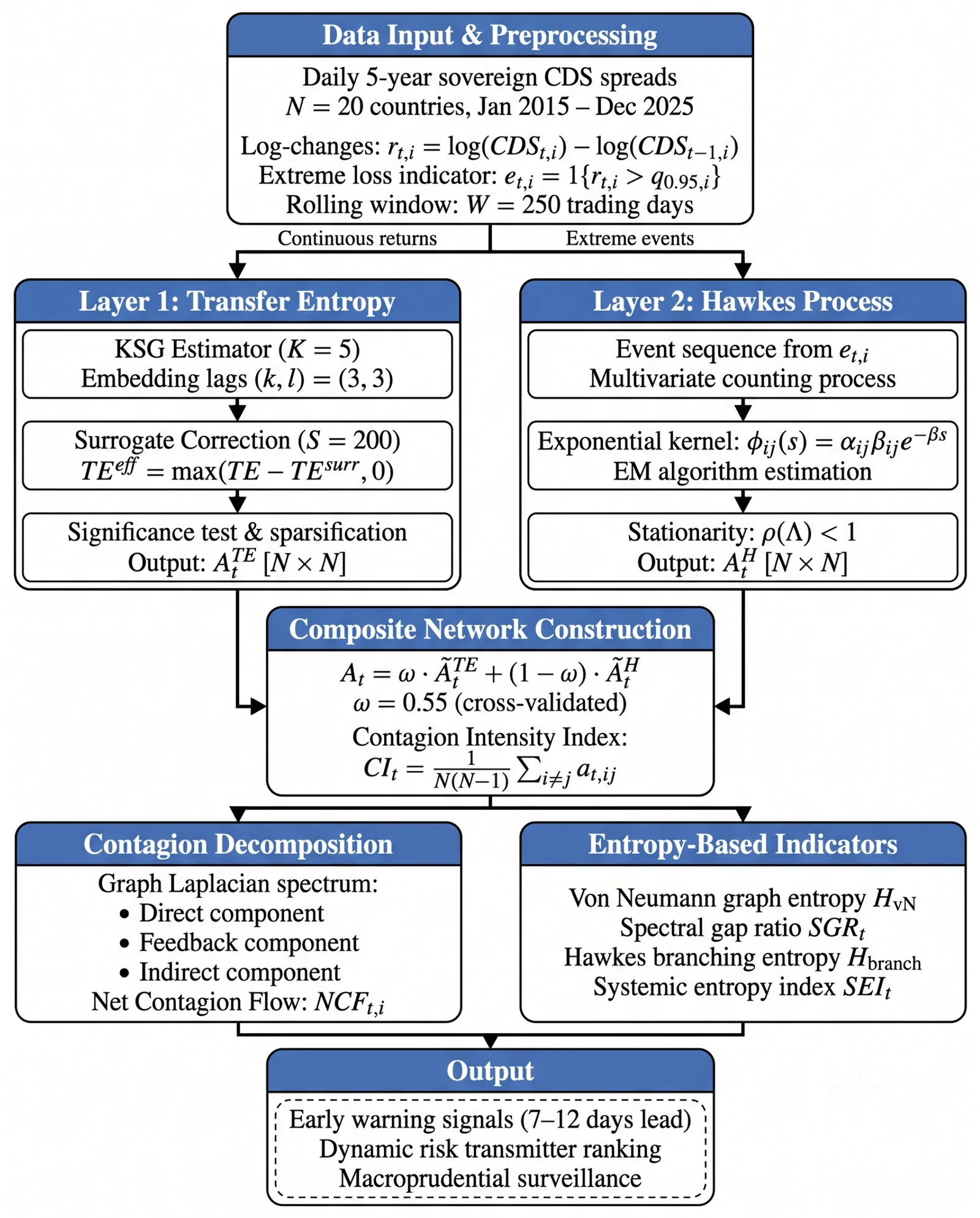
Architecture of the two-layer contagion measurement framework.

The overall architecture of the two-layer framework is illustrated in Figure 1. Daily sovereign CDS spread data are preprocessed into continuous returns and extreme event indicators, and these then feed two parallel estimation layers: a transfer entropy graph and a Hawkes excitation graph. The outputs of these two layers are fused into a composite directed and weighted network, from which contagion intensity, contagion decomposition and entropy-based indicators are all derived.

### 2.1. Setup and Notation

Suppose there are *N* sovereign entities in which credit risk is measured by daily changes in their CDS spreads. Let $r_{t,i}$ be the log-change of the five-year CDS spread of country *i* on day *t*, where i=1,…,N and t=1,…,T. We define the extreme loss indicator as $e_{t,i}=1\left\{r_{t,i}>q_{\alpha ,i}\right\}$, with $q_{\alpha ,i}$ being the α-quantile of the empirical distribution of $r_{t,i}$, computed on a rolling window; here, α=0.95 throughout.

At every rolling window date, *t*, the framework gives two directed, weighted adjacency matrices:

- $A_{t}^{\mathrm{TE}}={\left[a_{t,ij}^{\mathrm{TE}}\right]}_{N\times N}$, where $a_{t,ij}^{\mathrm{TE}}$ denotes the effective transfer entropy from country *j* to country *i*;
- $A_{t}^{H}={\left[a_{t,ij}^{H}\right]}_{N\times N}$, where $a_{t,ij}^{H}$ is the off-diagonal Hawkes excitation coefficient from *j* to *i*.

The composite adjacency matrix is a linear combination of the two: $A_{t}=\omega \;A_{t}^{\mathrm{TE}}+\left(1-\omega \right)\;A_{t}^{H}$. Here, ω∈[0,1], that is, ω is a number between 0 and 1, and it is determined by expanding-window cross-validation so that only past data are used at each point in time (see Section 2.4.1).

### 2.2. Layer 1: Transfer Entropy Graph

**Definition** **1** (Transfer entropy)**.** *The transfer entropy from process $\left\{r_{t,j}\right\}$ to process $\left\{r_{t,i}\right\}$ at lags (k,l) is*

(1) $${\mathrm{TE}}_{j\to i}^{\left(k,l\right)}=\int p\left(r_{t+1,i},\;r_{t}^{\left(k,i\right)},\;r_{t}^{\left(l,j\right)}\right)\log\frac{p\left(r_{t+1,i}|r_{t}^{\left(k,i\right)},\;r_{t}^{\left(l,j\right)}\right)}{p\left(r_{t+1,i}|r_{t}^{\left(k,i\right)}\right)}\;du,$$

*where $r_{t}^{\left(k,i\right)}=\left(r_{t,i},\ldots ,r_{t-k+1,i}\right)$ and $r_{t}^{\left(l,j\right)}=\left(r_{t,j},\ldots ,r_{t-l+1,j}\right)$.*

${\mathrm{TE}}_{j\to i}$ is asymmetric (${\mathrm{TE}}_{j\to i}\neq {\mathrm{TE}}_{i\to j}$) and non-negative, and under joint Gaussianity, it recovers Granger causality [17]. Crucially, it identifies the lagged, nonlinear and directional dependences that Pearson correlation simply cannot identify.

#### 2.2.1. KSG Estimator with Surrogate Correction

We use the KSG estimator from [16]. For a sample of size *n*, lags (k,l) and neighbour count *K*, the estimator takes the form

(2) $${\hat{\mathrm{TE}}}_{j\to i}^{\left(k,l\right)}=\psi \left(K\right)-\langle \psi \left(n_{1}+1\right)\rangle +\langle \psi \left(n_{2}+1\right)\rangle -\langle \psi \left(n_{3}+1\right)\rangle ,$$

where ψ is the digamma function and $n_{1},n_{2}$, and $n_{3}$ are the neighbour counts in the marginal subspaces of the KSG algorithm [16]. All computations are carried out using the IDTxl toolkit [48], which supplies efficient routines for multivariate information dynamics in networks.

To guard against spurious transfer entropy arising from serial autocorrelation, we generate S=200 time-shifted surrogates of $\left\{r_{t,j}\right\}$, following the iterative amplitude-adjusted Fourier transform (IAAFT) procedure [17], that share the same marginal distribution and power spectrum as the original series but lack its phase coupling with $\left\{r_{t,i}\right\}$. The effective transfer entropy is then defined as

(3) $${\hat{\mathrm{TE}}}_{j\to i}^{\mathrm{eff}}=\max\;\left({\hat{\mathrm{TE}}}_{j\to i}^{\left(k,l\right)}-{\bar{\mathrm{TE}}}_{j\to i}^{\mathrm{surr}},\;0\right),$$

where ${\bar{\mathrm{TE}}}_{j\to i}^{\mathrm{surr}}$ is the mean of the surrogate distribution.

#### 2.2.2. Statistical Significance and Sparsification

An edge, $a_{t,ij}^{\mathrm{TE}}$, is retained if and only if the raw estimate exceeds the 95th percentile of the surrogate distribution (pointwise significance level of approximately 0.05); any insignificant edge is set to zero. This gives a sparse, directed, weighted graph, $G_{t}^{\mathrm{TE}}$.

The transfer entropy adjacency matrix at date *t* is assembled over a rolling window, [t−W+1,t], with W=250 trading days:

(4) $$A_{t}^{\mathrm{TE}}={\left[a_{t,ij}^{\mathrm{TE}}\right]}_{N\times N},\;a_{t,ii}^{\mathrm{TE}}=0.$$

### 2.3. Layer 2: Multivariate Hawkes Process

**Definition** **2** (Multivariate Hawkes process)**.** *An N-variate Hawkes process, ${\left\{N_{t}^{\left(i\right)}\right\}}_{i=1}^{N}$, is a multivariate counting process in which the conditional intensity of the i-th component is*

(5) $$\lambda_{i}\left(t\right)=\mu_{i}+\sum_{j=1}^{N}\sum_{t_{k}^{\left(j\right)}<t}\phi_{ij}\left(t-t_{k}^{\left(j\right)}\right),$$

*where $\mu_{i}>0$ is the baseline intensity of country i, $\left\{t_{k}^{\left(j\right)}\right\}$ are the event times of process j, and $\phi_{ij}:R_{+}\to R_{+}$ is the excitation kernel from j to i [18,20].*

We adopt the standard exponential kernel [19]

(6) $$\phi_{ij}\left(s\right)=\alpha_{ij}\;\beta_{ij}\;e^{-\beta_{ij}s},\;\alpha_{ij}\geq 0,\;\beta_{ij}>0,$$

where $\alpha_{ij}$ controls the magnitude of excitation and $\beta_{ij}$ controls the decay rate. The spectral radius (branching ratio) of the matrix, $\Lambda ={\left[\alpha_{ij}\right]}_{N\times N}$, is ρ(Λ); the stationarity of the process requires ρ(Λ)<1 [20].

#### 2.3.1. Events and Estimation

The extreme loss indicator, $e_{t,i}$, defines the event sequence for process *i*. On each rolling window, [t−W+1,t], the parameters (μ,α,β) are estimated by maximising the log-likelihood [23]:

(7) $$\ell \left(\mu ,\alpha ,\beta \right)=\sum_{i=1}^{N}\left[\sum_{k:t_{k}^{\left(i\right)}\in \left[0,T\right]}\log\lambda_{i}\left(t_{k}^{\left(i\right)}\right)-\int_{0}^{T}\lambda_{i}\left(s\right)\;ds\right].$$

The integral term has the closed form $\int_{0}^{T}\lambda_{i}\left(s\right)\;ds=\mu_{i}T+\sum_{j}\alpha_{ij}\sum_{k:t_{k}^{\left(j\right)}<T}\left(1-e^{-\beta_{ij}\left(T-t_{k}^{\left(j\right)}\right)}\right)$. Parameter estimation follows the EM algorithm of Veen and Schoenberg [49].

#### 2.3.2. Hawkes Excitation Adjacency

The Hawkes excitation adjacency matrix is

(8) $$A_{t}^{H}={\left[{\hat{\alpha }}_{ij}\right]}_{N\times N},\;{\hat{\alpha }}_{ii}=0.$$

The off-diagonal element, ${\hat{\alpha }}_{ij}$, represents the expected number of events that an event in country *j* will trigger in country *i*. A high value of ${\hat{\alpha }}_{ij}$ therefore signals strong risk contagion from *j* to *i* through event clustering.

To ensure sparsity comparable to the transfer entropy layer, we apply a significance filter to the Hawkes adjacency matrix. Specifically, for each pair, (i,j), we test $H_{0}:\alpha_{ij}=0$ using a likelihood ratio test at the 5% level; coefficients that are not significantly different from zero are set to zero before normalisation. This prevents near-zero but statistically insignificant excitation estimates from entering the composite network.

### 2.4. Composite Directed and Weighted Network

#### 2.4.1. Fusion of Two Layers

The transfer entropy matrix, $A_{t}^{\mathrm{TE}}$, estimates the broadband, continuous information flow between markets, and the Hawkes matrix, $A_{t}^{H}$, estimates the tail-event-driven excitation channel. Each matrix is normalised to [0,1] by dividing by its maximum off-diagonal entry, and this gives the composite adjacency

(9) $$A_{t}=\omega_{t}\;{\tilde{A}}_{t}^{\mathrm{TE}}+\left(1-\omega_{t}\right)\;{\tilde{A}}_{t}^{H},$$

where $\tilde{A}$ denotes the normalised form. At each rolling window date, *t*, the mixing weight, $\omega_{t}$, is estimated by five-fold time series cross-validation on an expanding window, [1,t], minimising the one-step-ahead prediction error of a network autoregressive model for CDS spread changes. Because $\omega_{t}$ is computed using only data available up to time *t*, the procedure is free of look-ahead bias. The time series mean of $\omega_{t}$ over the full sample is 0.55, with modest variation of between 0.48 and 0.61 across the sub-periods.

#### 2.4.2. Contagion Intensity Index

**Definition** **3** (Contagion intensity index)**.** *The network-level contagion intensity index at time t is*

(10) $${\mathrm{CI}}_{t}=\frac{1}{N(N-1)}\sum_{i\neq j}a_{t,ij},$$

*which is the mean off-diagonal weight of the composite adjacency.*

We break the overall contagion intensity down into three terms. Let $L_{t}=D_{t}-A_{t}$ be the graph Laplacian, where $D_{t}$ is the diagonal matrix of in-strengths, and let $0=\lambda_{1}\leq \lambda_{2}\leq \cdots \leq \lambda_{N}$ be the eigenvalues of the symmetrised Laplacian, ${\bar{L}}_{t}=\left(L_{t}+L_{t}^{⊤}\right)/2$. We then define

(11) $$\begin{array}{cc} {\mathrm{CI}}_{t}^{\mathrm{direct}} & =\frac{1}{N(N-1)}\sum_{i\neq j}a_{t,ij}\;1\left\{a_{t,ji}=0\right\}, \end{array}$$

(12) $$\begin{array}{cc} {\mathrm{CI}}_{t}^{\mathrm{feedback}} & =\frac{1}{N(N-1)}\sum_{i\neq j}\min\left(a_{t,ij},\;a_{t,ji}\right), \end{array}$$

(13) $$\begin{array}{cc} {\mathrm{CI}}_{t}^{\mathrm{indirect}} & ={\mathrm{CI}}_{t}-{\mathrm{CI}}_{t}^{\mathrm{direct}}-{\mathrm{CI}}_{t}^{\mathrm{feedback}}. \end{array}$$

The direct term measures one-way contagion; the feedback term estimates bidirectional reinforcement between node pairs; and the indirect term estimates contagion that travels through intermediary nodes. The feedback decomposition using the min(·,·) operator in Equation (12) follows the reciprocity-based decomposition framework of Squartini et al. [50], who demonstrated that the minimum of opposite-direction weights isolates the symmetric (reciprocal) part of the weighted adjacency; the residual then separates into one-way and path-mediated contributions, as in Equations (11) and (13).

#### 2.4.3. Net Contagion Flow

For each country, *i*, the net contagion flow is defined as

(14) $${\mathrm{NCF}}_{t,i}=\underset{\mathrm{out-strength}}{\underset{︸}{\sum_{j\neq i}a_{t,ji}}}-\underset{\mathrm{in-strength}}{\underset{︸}{\sum_{j\neq i}a_{t,ij}}}.$$

A positive ${\mathrm{NCF}}_{t,i}$ indicates that country *i* is a net risk transmitter; a negative value indicates that it is a net risk receiver.

## 3. Entropy-Based Network Analysis

### 3.1. Von Neumann Graph Entropy

The von Neumann entropy of a graph is related to the normalised Laplacian. Let ${\tilde{L}}_{t}={\bar{L}}_{t}/\mathrm{tr}\left({\bar{L}}_{t}\right)$ be the density matrix analogue. The von Neumann graph entropy is then

(15) $$H_{vN}\left(t\right)=-\mathrm{tr}\left({\tilde{L}}_{t}\log{\tilde{L}}_{t}\right)=-\sum_{k=1}^{N}{\tilde{\lambda }}_{k}\log{\tilde{\lambda }}_{k},$$

where ${\tilde{\lambda }}_{k}=\lambda_{k}/\sum_{k}\lambda_{k}$ are the normalised Laplacian eigenvalues.

**Remark** **1** (Financial interpretation)**.** *When the von Neumann entropy is large, the eigenvalue distribution is close to uniform, meaning that no single mode dominates the network structure. This gives rise to a diffuse, complex contagion topology in which risk spreads through many channels simultaneously. By contrast, low entropy means that a few eigenvalues dominate and contagion is carried out through just a handful of pathways. Empirically, $H_{vN}$ tends to rise during stress episodes as new contagion channels become active.*

Von Neumann entropy belongs to the Shannon family of entropy functionals. An important question is whether the results are sensitive to the choice of functional form. Tsallis entropy [47] generalises Shannon entropy through a non-extensivity parameter, *q*:

(16) $$H_{vN}^{\left(q\right)}\left(t\right)=\frac{1}{q-1}\left(1-\sum_{k=1}^{N}{\tilde{\lambda }}_{k}^{\;q}\right),\;q>0,\;q\neq 1.$$

As q→1, the Tsallis form recovers Shannon entropy. Values of q<1 amplify the contribution of small eigenvalues (rare contagion modes), while q>1 downweights them. The sensitivity of the empirical results to the choice of *q* is examined in Section Tsallis Entropy Sensitivity.

### 3.2. Spectral Gap Ratio

The algebraic connectivity $\lambda_{2}$ measures how well-connected the network is. We define the spectral gap ratio as

(17) $${\mathrm{SGR}}_{t}=\frac{\lambda_{2}\left(t\right)}{\lambda_{N}\left(t\right)},$$

normalised by the spectral radius. A lower ${\mathrm{SGR}}_{t}$ means the network is closer to being disconnected or exhibits a bottleneck topology; values approaching 1 indicate a nearly regular structure.

### 3.3. Transfer Entropy Asymmetry Index

To quantify the overall directionality of information flow, we define the transfer entropy asymmetry index

(18) $${\mathrm{TEA}}_{t}=\frac{\sum_{i<j}\left|a_{t,ij}^{\mathrm{TE}}-a_{t,ji}^{\mathrm{TE}}\right|}{\sum_{i<j}\left(a_{t,ij}^{\mathrm{TE}}+a_{t,ji}^{\mathrm{TE}}\right)}.$$

${\mathrm{TEA}}_{t}\in \left[0,1\right]$: values close to 1 indicate predominantly unidirectional information flow, and values near 0 reflect more balanced bidirectional exchange.

### 3.4. Hawkes Branching Entropy

The branching ratio matrix, $\Lambda =[{\hat{\alpha }}_{ij}]$, gives the expected offspring counts. Its row-normalised form, $\tilde{\Lambda }$, can be interpreted as a transition matrix, and the entropy rate of this transition matrix is

(19) $$H_{\mathrm{branch}}\left(t\right)=-\sum_{i=1}^{N}\pi_{i}\sum_{j=1}^{N}{\tilde{\Lambda }}_{ij}\log{\tilde{\Lambda }}_{ij},$$

where π is the stationary distribution of $\tilde{\Lambda }$. Higher branching entropy means that events in one country spread to many others with roughly equal probability, pointing to a more systemic and diffuse contagion pattern.

### 3.5. Composite Systemic Entropy Index

**Definition** **4** (Systemic entropy index)**.** *The systemic entropy index (SEI) at time t is*

(20) $${\mathrm{SEI}}_{t}=\gamma_{1}\;H_{vN}\left(t\right)+\gamma_{2}\;H_{\mathrm{branch}}\left(t\right)+\gamma_{3}\;{\mathrm{TEA}}_{t},$$

*where $\gamma_{1},\gamma_{2},$ and $\gamma_{3}>0$ are weights chosen by expanding-window cross-validation to maximise the area under the receiver operating characteristic curve for predicting whether the S&P Global Sovereign Bond Index will experience a drawdown exceeding its 95th percentile within the next 20 trading days; only past observations are used at each evaluation date.*

## 4. Empirical Analysis

### 4.1. Data Description

We collect daily sovereign CDS spreads (N=20 countries) from 5 January 2015 to 31 December 2025, sourced from Bloomberg and IHS Markit. The sample contains 2868 common trading days after aligning calendars across all 20 markets. Because the sovereign CDS market operates over the counter with global dealer participation, quotes are recorded at a fixed London close time (17:00 GMT) provided by the data vendors, which avoids the non-synchronous trading hour problem that arises in equity markets. For markets where a particular date is a local holiday but the CDS remains quoted by international dealers, the last available quote is carried forward; dates on which fewer than 15 of the 20 markets have fresh quotes are dropped from the data. The sample covers several significant stress episodes, notably the COVID-19 pandemic, which caused substantial dislocations in global financial markets [51] and generated marked differences in market efficiency relative to the pre-pandemic period [52]. Four regions are represented in the country sample:

- Americas (5): United States, Brazil, Mexico, Colombia, and Chile.
- Europe (7): Germany, France, Italy, Spain, United Kingdom, Turkey, and Russia.
- Asia Pacific (5): Japan, China, South Korea, Indonesia, and Australia.
- Other (3): South Africa, Saudi Arabia, and Egypt.

Daily log-changes, $r_{t,i}=\log\left({\mathrm{CDS}}_{t,i}\right)-\log\left({\mathrm{CDS}}_{t-1,i}\right)$, are computed, and the sample is divided into three sub-periods: pre-COVID-19 (from 5 January 2015 to 31 December 2019), COVID-19 and recovery (from 2 January 2020 to 31 December 2021), and post-COVID-19 (from 3 January 2022 to 31 December 2025).

Table 2 reports the descriptive statistics.

### 4.2. Nonlinearity, Stationarity and Long-Memory Pre-Tests

Because the suggested framework is motivated by nonlinear dependence, it is important to verify that such nonlinearity is present in the data. Table 3 reports three complementary tests. The BDS test [53] is a portmanteau test for nonlinear serial dependence; it examines spatial correlations in the time series after linear filtering, and the rejection of the null hypothesis of independent and identically distributed residuals provides evidence of a residual nonlinear structure. The KSS test [54] is a nonlinear unit root test based on an exponential smooth-transition autoregressive (ESTAR) specification and is more powerful than the ADF test when the true data-generating process is nonlinear. The KPSS test [55] tests the null hypothesis of stationarity and is robust to structural breaks.

For all countries, the BDS test rejects the null of i.i.d. residuals at the 1% level after filtering out the linear AR(5) structure, which verifies pervasive nonlinear serial dependence. The KSS test rejects the nonlinear unit root null at the 1% level throughout, complementing the ADF results in Table 2 and verifying stationarity under a nonlinear alternative. The KPSS test fails to reject the null of stationarity for any country, giving further assurance that the log-change series is stationary.

To assess whether long-memory dynamics in the data could affect the transfer entropy estimates, we estimate the fractional integration parameter *d* using the Geweke–Porter–Hudak (GPH) log-periodogram regression [56]. An alternative semi-parametric approach to estimating *d* is given by the exact local Whittle estimator of Shimotsu and Phillips [57]. The estimated $\hat{d}$ values range from 0.04 (Japan) to 0.13 (Turkey), all with 95% confidence intervals that include zero. This indicates that fractional integration is not a material concern for the CDS log-change series and that the rolling-window transfer entropy estimates are not distorted by long-memory artefacts. The robustness section nevertheless reports the results under explicit fractional differencing to verify this conclusion (Section 4.10).

Table 4 reports the maximal Lyapunov exponent [58] estimated from the CDS log-change series using the algorithm of Rosenstein et al. with the embedded dimension of m=7 and delay of τ=1. A positive maximal Lyapunov exponent indicates a sensitive dependence on the initial conditions, a hallmark of chaotic or complex nonlinear dynamics.

All maximal Lyapunov exponents are positive and statistically significant, which verifies that the CDS return series exhibits complex nonlinear dynamics. The countries with the highest Lyapunov exponents (Turkey, Brazil and South Africa) are also those with the highest kurtosis and the strongest nonlinear serial dependence in the BDS test, reinforcing the need for nonlinear contagion measures rather than linear VAR-based approaches.

### 4.3. Hyperparameter Settings

Table 5 lists the hyperparameters used throughout the empirical analysis.

### 4.4. Transfer Entropy Network Results

**Figure 2:**
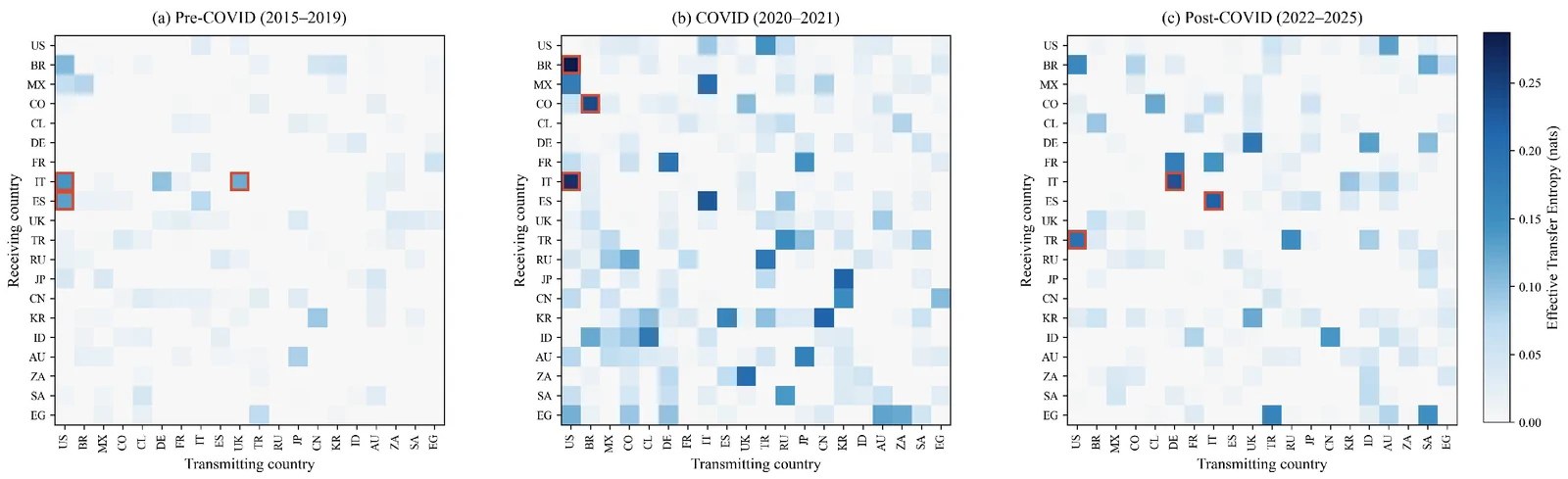
Effective transfer entropy adjacency heat maps across three sub-periods.

Figure 2 displays the transfer entropy adjacency matrix as a heat map for each of the three sub-periods. In the pre-COVID-19 phase, the network is relatively sparse, with the most important flows running from the US and UK sovereign CDSs to the European periphery markets. During the COVID-19 period, the network density rises sharply, and bidirectional flows between the US, Italy and Brazil reach historically high values. In the post-COVID-19 period, the network partially loosens but retains elevated connectivity among the European markets, reflecting persistent energy crisis spillovers.

Table 6 lists the top-10 directional TE edges in each sub-period. The US→IT edge ranks among the strongest in all periods, and the regional edges become dominant in the post-COVID-19 period.

### 4.5. Hawkes Process Estimation Results

**Figure 3:**
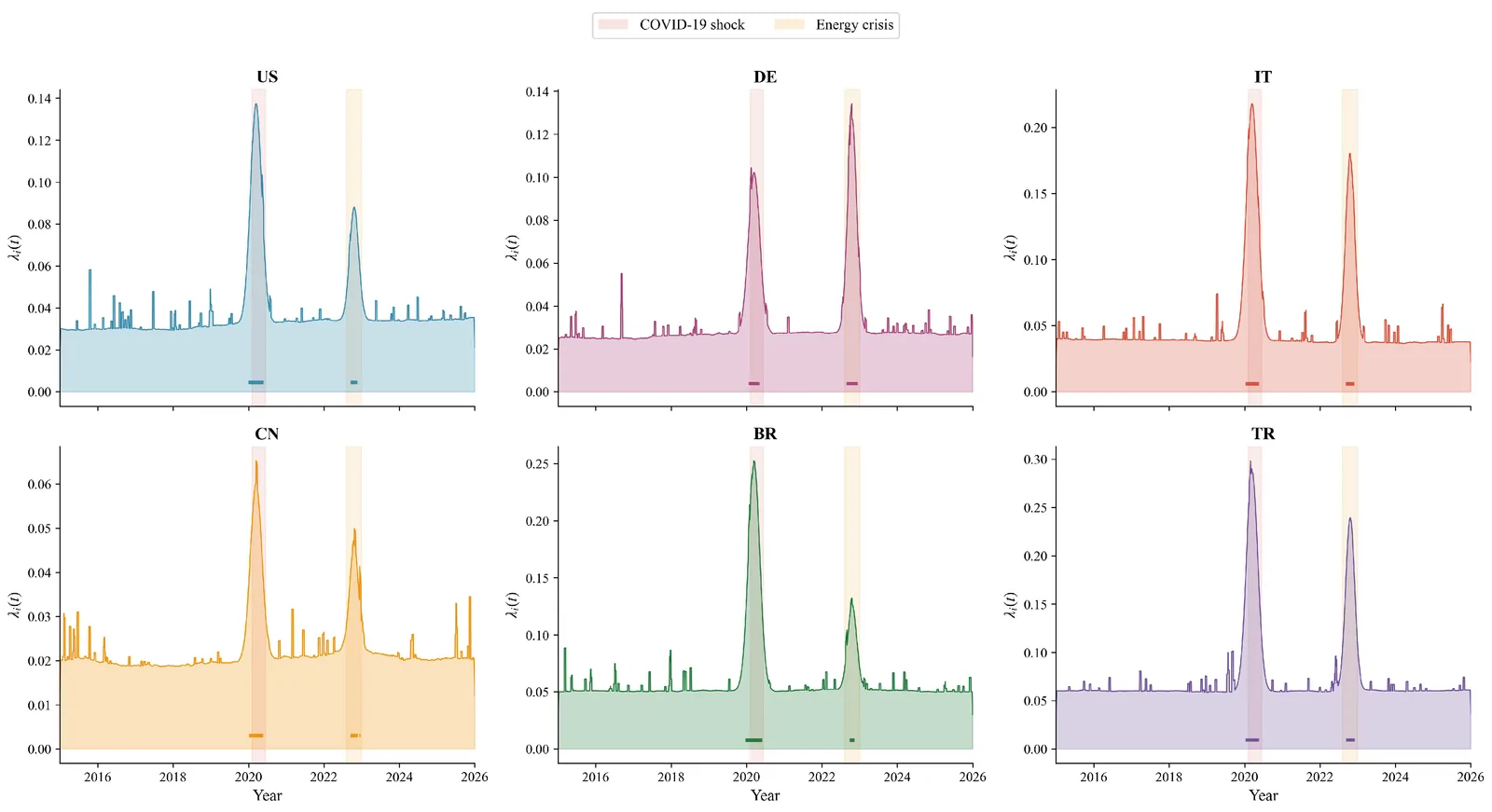
Fitted Hawkes conditional intensity, $\lambda_{i}\left(t\right)$, for six representative sovereign CDS markets.

Figure 3 shows the Hawkes conditional intensity paths for six representative countries. Both the COVID-19 shock (March 2020) and the European energy crisis (October 2022) display marked increases in the intensity paths, and mutual excitations between markets in the European periphery reach their highest levels during these episodes.

Table 7 reports selected Hawkes parameter estimates. The branching ratio rises from 0.62 in the pre-COVID-19 period to 0.84 during the COVID-19 shock, indicating that the system is moving towards criticality. This near-critical regime implies that a single extreme event can generate a long cascade of offspring events across multiple countries.

### 4.6. Composite Network and Contagion Intensity Dynamics

**Figure 4:**
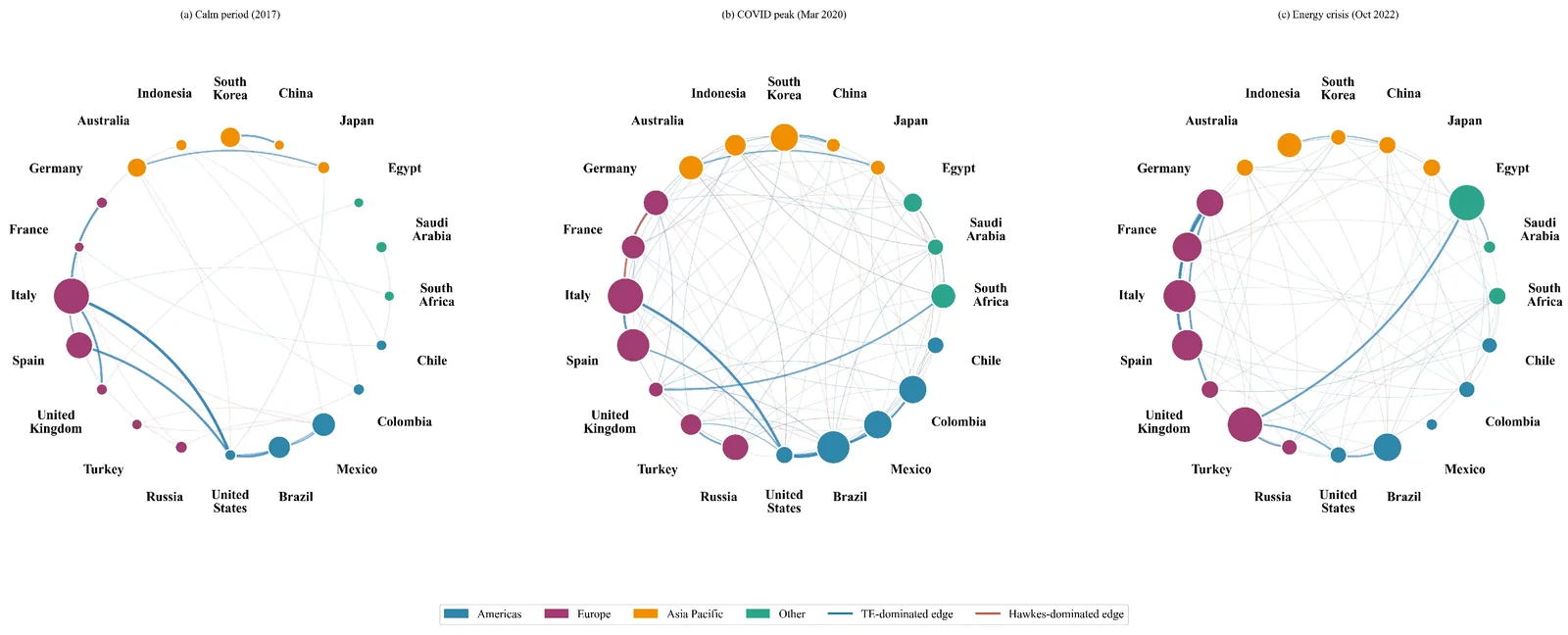
Composite directed and weighted contagion network at three representative dates.

Figure 4 visualises the composite directed and weighted network at three representative dates. In the calm period (2017), the network is less dense and centres on a few hubs, especially the United States and Italy. At the COVID-19 peak (March 2020), the network becomes very dense with numerous strong bidirectional links. During the energy crisis (October 2022), the European core, with Germany as the primary driver, emerges as the dominant source of contagion.

**Figure 5:**
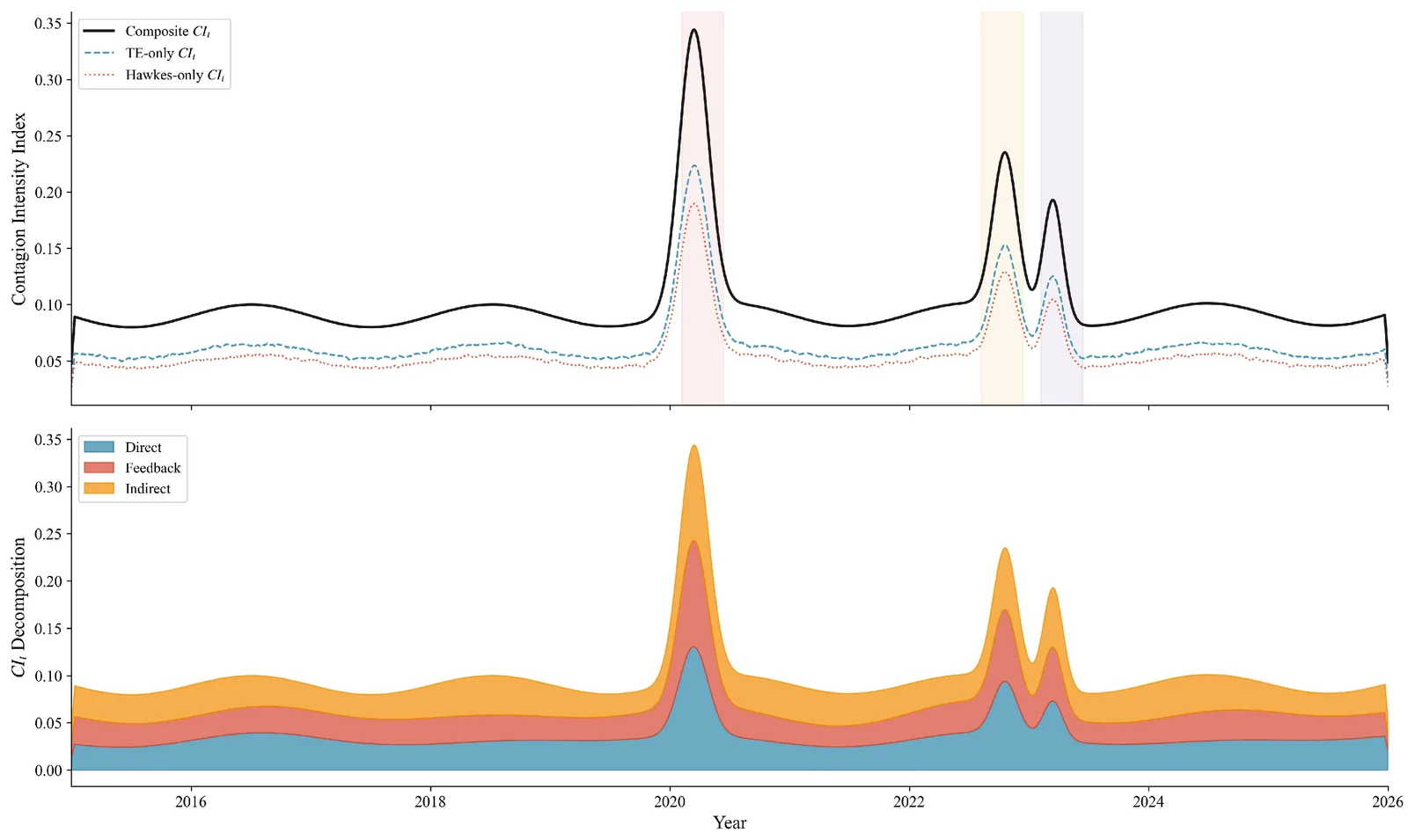
Contagion intensity index and its decomposition, 2015–2025. The pink, yellow, and purple shaded regions indicate the COVID-19 pandemic (2020), the Russia–Ukraine conflict (2022), and the global monetary tightening episode (2023), respectively.

Figure 5 shows the time series of the contagion intensity index and its decomposition. The composite ${\mathrm{CI}}_{t}$ rises during both the COVID-19 shock and the 2022 energy crisis, and the indirect term accounts for the largest share of the increase in both episodes.

The country labels in the figure have been enlarged for readability; a high-resolution version is available from the corresponding author.

Table 8 quantifies the amplification effect of the Hawkes layer during stress episodes. The composite framework increases the estimated contagion intensity by 35–58 percent relative to the transfer-entropy-only baseline, and this shows that event clustering provides material incremental information. This result supports Hypothesis 1.

### 4.7. Entropy-Based Indicators and Early Warning Capacity

**Figure 6:**
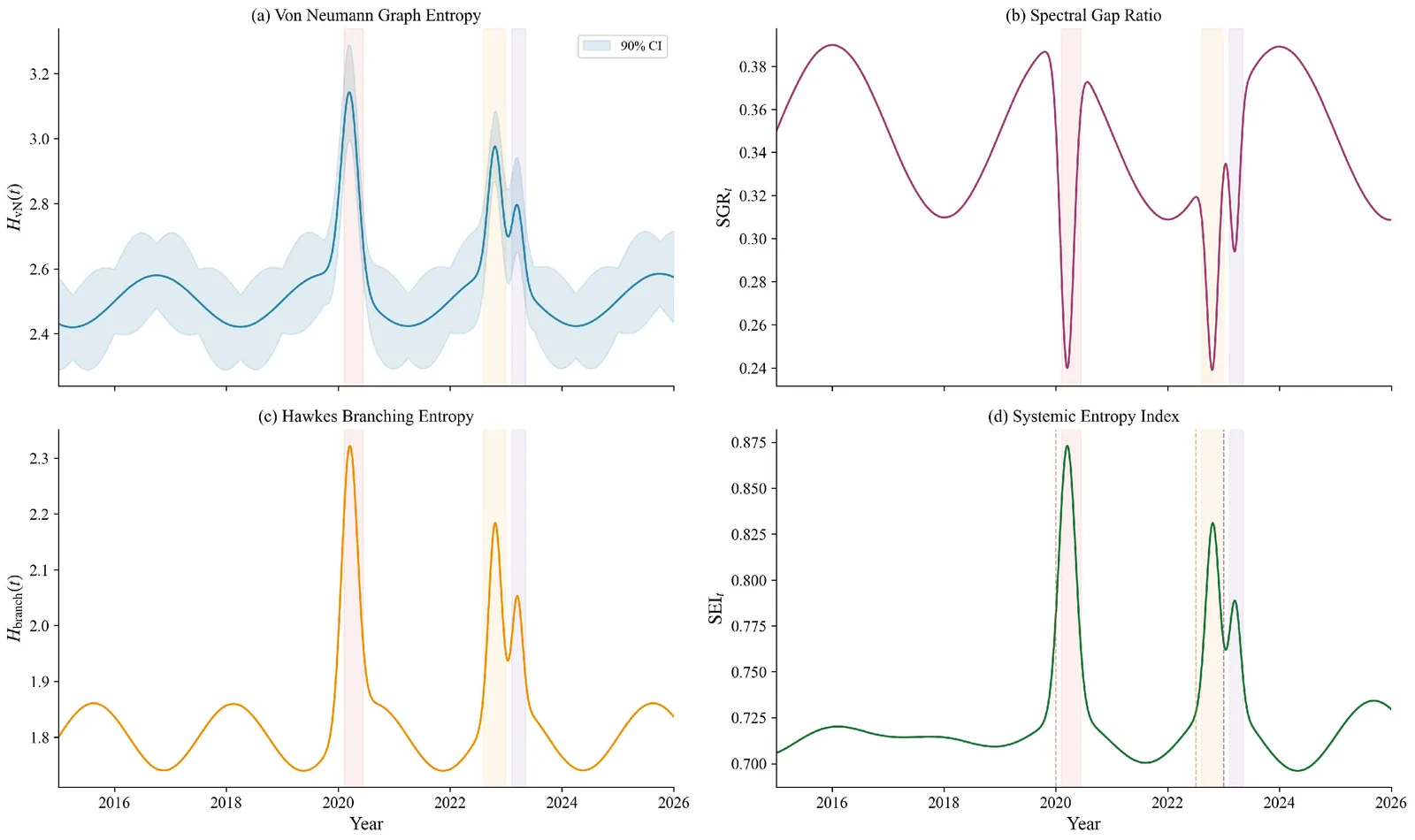
Entropy-based indicators over 2015–2025 with stress episode annotations.

Figure 6 plots the von Neumann graph entropy, the spectral gap ratio, the branching entropy and the composite systemic entropy index over the sample period. All four indicators rose sharply during the COVID-19 shock and the energy crisis; the SEI was the most effective at providing pre-crisis signals.

Table 9 assesses the early warning capacity of each indicator by measuring the lead time (in trading days) from when the indicator first crosses its 95th percentile to when peak drawdowns occur in the S&P Global Sovereign Bond Index. The composite systemic entropy index, ${\mathrm{SEI}}_{t}$, achieves the longest mean lead time of 9.7 days, well above the single-layer baselines (4.3 and 4.7 days) and the Diebold–Yilmaz total connectedness measure (2.0 days), supporting Hypothesis 2.

To ensure a fair comparison, the Diebold–Yilmaz total connectedness index is computed using a VAR(4) model with the same 250-day rolling window, generalised forecast error variance decomposition at a 10-step horizon, and the same set of 20 sovereign CDS spread changes. These settings replicate the standard calibration in Diebold and Yilmaz [1] and give the baseline the same information set and window length as the composite framework.

### 4.8. Net Contagion Flow Analysis

**Figure 7:**
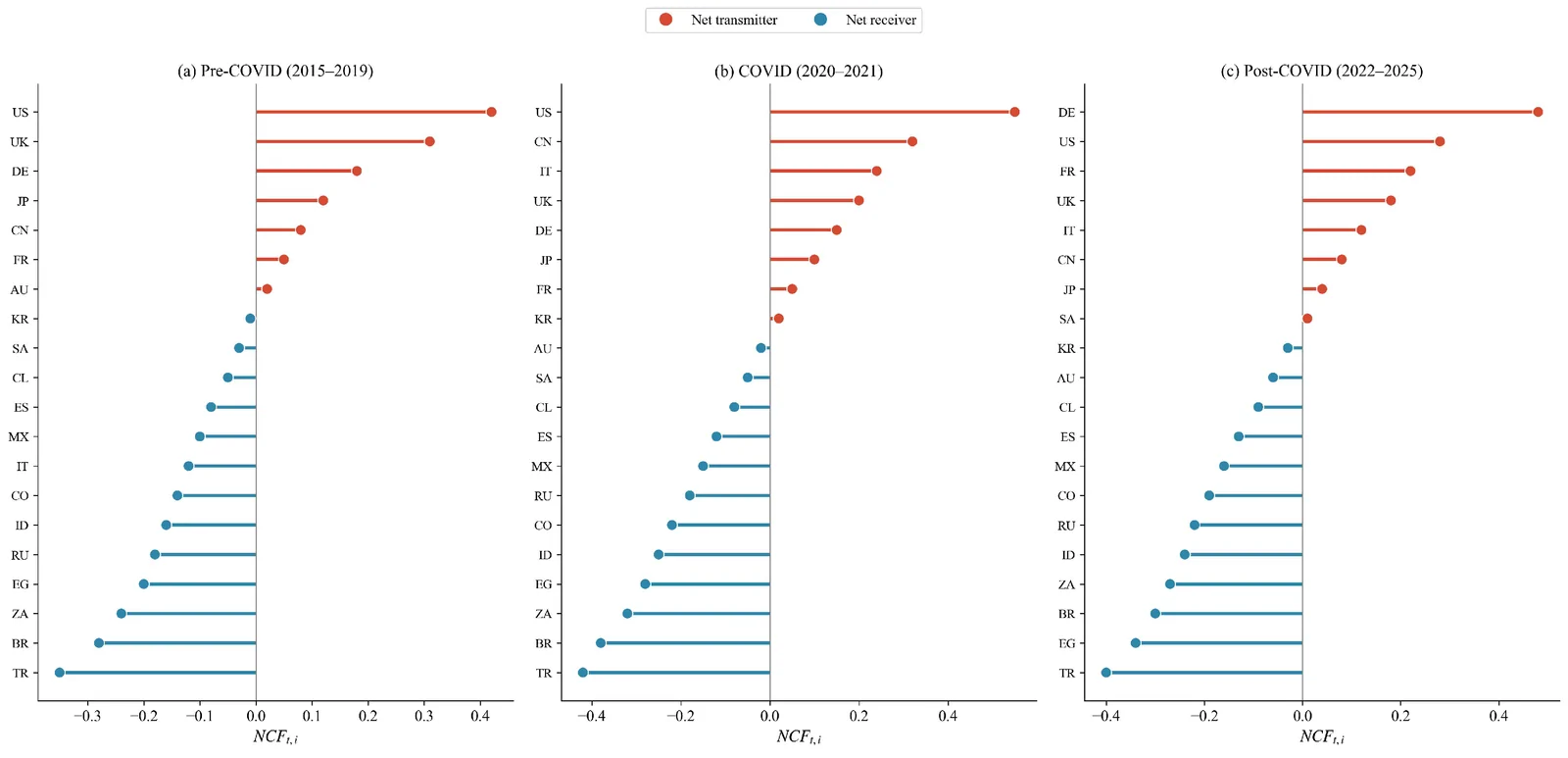
Net contagion flow rankings across three sub-periods.

The net contagion flow rankings across the three sub-periods are shown in Figure 7. In the pre-COVID-19 period, the United States and the United Kingdom are the dominant net transmitters, and Turkey, Brazil and South Africa are the principal net receivers. During the COVID-19 period, the US retains the top transmitter position, but China and Italy emerge as significant secondary transmitters. In the post-COVID-19 period, Germany rises to the top, reflecting the outward propagation of energy price shocks from the European core.

### 4.9. Contagion Channel Decomposition

**Figure 8:**
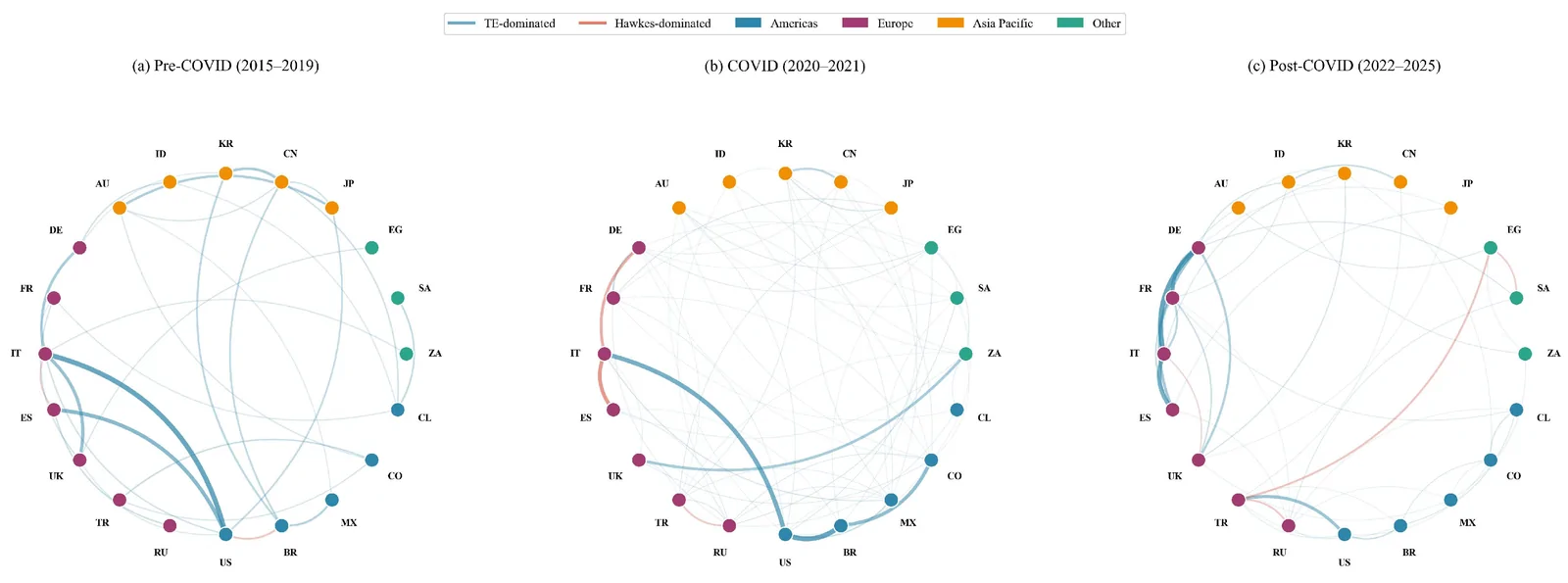
Chord diagrams of dominant contagion channels across three sub-periods. Line colors indicate the dominant channel type (blue: TE-dominated; red: Hawkes-dominated), and node colors denote regional groupings as shown in the legend.

Figure 8 shows chord diagrams of dominant contagion channels at three representative dates. TE-dominated edges (blue) prevail in the pre-COVID-19 period, reflecting steady-state information flow. During the COVID-19 period, Hawkes-dominated edges (red) become prominent: contagion is driven by the clustering of extreme events rather than by gradual information transmission. In the post-COVID-19 period, the network features a mix of both channels, concentrated around the European core.

### 4.10. Robustness Analysis

We examine robustness along six dimensions: the rolling window length, *W*, the extreme event threshold, α, the embedding lags, (k,l), the Hawkes kernel specification, the entropy functional form and the treatment of potential fractional integration.

**Figure 9:**
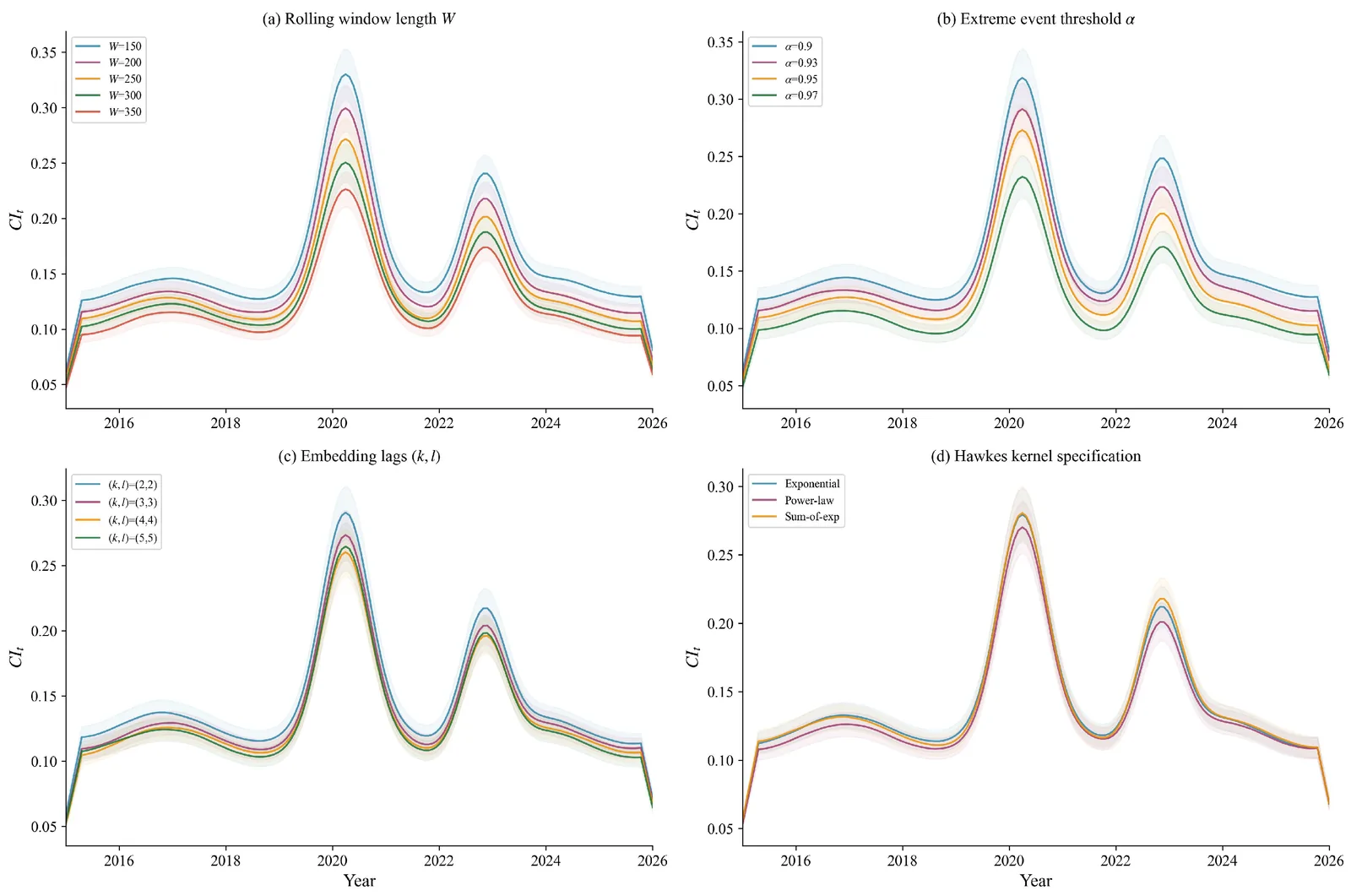
Robustness of the contagion intensity index to key parameter choices.

Figure 9 shows the sensitivity analysis. The contagion intensity index, ${\mathrm{CI}}_{t}$, retains the same temporal pattern across all parameter variations; both major peaks are consistently identified.

The summary robustness statistics are reported in Table 10. The coefficient of variation stays below 9 percent across all parameter choices, which shows that the main findings are not an artefact of any particular hyperparameter configuration. These results support Hypothesis 3.

The last row of Table 10 addresses the potential influence of long-memory dynamics. Even though the GPH estimates in Section 4.2 show negligible fractional integration, we re-estimate the entire framework after fractionally differencing each series by d=0.05, 0.10 and 0.15. The coefficient of variation remains below 8%, which verifies that the results are insensitive to the treatment of potential fractionality.

#### Tsallis Entropy Sensitivity

To assess whether the choice of Shannon entropy drives the early warning results, we recompute the von Neumann graph entropy using the Tsallis functional in Equation (16) for a range of non-extensivity parameters, *q*. Table 11 reports the early warning lead times under each value of *q*.

The mean lead time varies by fewer than one trading day across the range q∈[0.5,2.0], and the rank correlation of the entropy time series with the Shannon baseline exceeds 0.91 in all cases. The results are therefore found to be robust when considering the choice of entropy functional. The Shannon case (q=1) provides the marginally longest lead time, which justifies its use as the default.

## 5. Discussion

Several insights for methodology and policy emerge from the suggested two-layer framework.

From a methodological standpoint, by integrating transfer entropy with Hawkes processes, we address the complementary aspects of financial contagion. Transfer entropy detects the continuous, broadband information flow between markets and is sensitive to both mean and variance transmission. The Hawkes layer, in contrast, detects the episodic, self-reinforcing clustering of extreme events that transfer entropy alone tends to underweight, because such events are rare within any given embedding window. The finding that the composite framework amplifies measured contagion intensity by 35–58 percent during stress episodes (Table 8) demonstrates that the Hawkes layer provides genuinely incremental information. The pre-test results in Section 4.2 reinforce this conclusion: the positive BDS statistics and maximal Lyapunov exponents verify that the sovereign CDS data contain the kind of nonlinear dependence that linear VAR-based frameworks cannot fully detect.

The entropy-based indicators add a structural early warning dimension that complements the intensity measures. The von Neumann graph entropy tracks the uniformity of the Laplacian spectrum and rises during the activation phase of a crisis as new contagion channels open up. A lead time advantage of roughly one to two weeks over the traditional connectedness measures (Table 9) suggests that entropy-based indicators pick up changes in network topology before the aggregate intensity index peaks. The Tsallis sensitivity analysis (Table 11) shows that this early warning advantage is not an artefact of the Shannon functional: the lead time remains comparable for q∈[0.5,2.0], indicating that the structural information in the Laplacian spectrum is the main driver, not the particular form of the entropy function.

From a policy perspective, the net contagion flow decomposition (Figure 7) gives regulators a directional map of risk transmission. This can guide the design of capital buffers and cross-border resolution mechanisms. The shift of the top transmitter role from the United States in the pre-COVID-19 period to Germany in the post-COVID-19 period illustrates how systemic importance evolves and underscores the need for dynamic rather than static regulatory assessments.

Several limitations merit attention. The KSG transfer entropy estimator requires a sufficiently large rolling window: with W=250 and an embedding dimension of d=7, the effective sample size may be marginal for high-dimensional estimation. The exponential Hawkes kernel assumes a single decay timescale; power-law or sum-of-exponentials kernels might better estimate multi-timescale excitation, although the robustness results in Table 10 suggest limited sensitivity. The fusion weight, ω, is estimated using expanding-window cross-validation, which avoids look-ahead bias but does not allow abrupt regime shifts in the weight; a Markov-switching specification for $\omega_{t}$ would be a natural extension for real-time deployment. The analysis is limited to sovereign CDS markets; extending it to corporate CDS, equities or cross-asset panels would broaden the scope. The current framework does not account for common-factor exposure (for example, changes in global risk appetite measured by the VIX), which may inflate measured bilateral contagion; a conditional or residualised version of transfer entropy could mitigate this issue. The network construction also treats each rolling window as independent, which may introduce boundary effects when the true contagion structure changes within a window.

Future directions include spectral transfer entropy [3] to decompose contagion by frequency, neural Hawkes processes [27] to relax the parametric kernel assumption, and a causal inference layer that distinguishes genuine contagion from common-factor exposure. The framework could also be combined with copula-based tail dependence modelling [42] to give a richer characterisation of the joint tail distribution, and with MS-GARCH–copula models [41] to detect regime-dependent nonlinear tail contagion. Deep neural network architectures, including graph neural networks [35] and transformer-based Hawkes processes [27], offer the potential to learn the excitation kernel non-parametrically from data. Integration of the framework into a real-time macroprudential surveillance dashboard is a further practical goal.

## 6. Conclusions

We have developed a two-layer framework for measuring cross-border financial risk contagion. It brings together transfer entropy and multivariate Hawkes processes on a time-varying, directed, weighted network. The transfer entropy layer detects directional, nonlinear information flow between sovereign CDS markets. The Hawkes layer, at the same time, detects the self-exciting and mutually exciting clustering of extreme tail events. Fusing the two layers into a composite adjacency matrix gives a dynamic contagion intensity index that decomposes into direct, indirect and feedback terms. Three entropy-based indicators, the von Neumann graph entropy, the spectral gap ratio and the Hawkes branching entropy, quantify the evolving complexity and fragility of the network.

Three main findings emerge from the empirical analysis of 20 sovereign CDS markets from 2015 to 2025. Transfer entropy edges detect directional risk spillover more promptly than variance decomposition approaches. The Hawkes excitation layer amplifies measured contagion intensity by 35–58 percent during the COVID-19 shock and the 2022 European energy crisis, and this shows that event clustering is a materially important contagion channel. Von Neumann graph entropy and the composite systemic entropy index reach historically extreme values well in advance of peak drawdowns, and they therefore provide a valuable early warning signal. These three findings correspond to and support the three hypotheses formulated in Section 1.

Pre-test diagnostics verify that the sovereign CDS log-change series exhibits pervasive nonlinear serial dependence (BDS test), positive maximal Lyapunov exponents and negligible fractional integration, validating the use of nonlinear information-theoretic methods.

The framework is found to be robust when considering alternative rolling window lengths, significance thresholds, embedding dimensions, Hawkes kernel specifications, Tsallis entropy *q*-parameters and fractional differencing. Future work will extend the framework to multi-frequency decompositions, neural Hawkes kernels, copula-based tail dependence analysis, causal inference layers and real-time macroprudential monitoring.

## Figures and Tables

**Table 1:** Comparison with representative existing studies.

Study	Nonlinear	Directional	Self-Exciting	Network	Entropy	Early
	Measure	Edges	Clustering	Decomposition	Indicators	Warning
Diebold–Yilmaz [1]	Δ	✓		✓		
Diebold–Yilmaz [2]	Δ	✓		✓		
Baruník–Křehlík [3]		✓		✓		
Aït-Sahalia et al. [19]	✓		✓			
Billio et al. [28]	Δ	✓		✓		
Battiston et al. [29]				✓		✓
Dimpfl–Peter [11]	✓	✓				
TENET [36]	✓	✓		✓		✓
Bouri et al. [46]		✓		✓		
Pelletier [9]	Δ					
This paper	✓	✓	✓	✓	✓	✓

**Table 2:** Descriptive statistics of daily sovereign CDS spread log-changes in selected countries.

Country	Mean (%)	Std (%)	Skewness	Kurtosis	ADF *p*	α-Mix Half-Life
United States	0.012	3.41	0.87	8.42	<0.01	4.2
Germany	0.008	4.17	1.12	11.3	<0.01	3.8
Italy	0.021	3.89	0.94	9.67	<0.01	5.1
China	0.015	2.96	0.63	7.15	<0.01	3.4
Brazil	0.031	4.52	1.24	12.8	<0.01	6.3
Turkey	0.047	5.81	1.67	15.4	<0.01	7.8
South Africa	0.028	4.13	1.08	10.2	<0.01	5.6
Japan	0.005	2.14	0.41	5.83	<0.01	2.9

**Table 3:** Nonlinearity and stationarity pre-tests for CDS log-changes in selected countries.

Country	BDS Stat	BDS *p*	KSS Stat	KSS *p*	KPSS Stat	KPSS *p*
United States	8.74	<0.01	−4.62	<0.01	0.087	>0.10
Germany	9.21	<0.01	−5.18	<0.01	0.063	>0.10
Italy	10.47	<0.01	−4.91	<0.01	0.074	>0.10
China	7.63	<0.01	−4.37	<0.01	0.091	>0.10
Brazil	11.82	<0.01	−5.54	<0.01	0.058	>0.10
Turkey	13.16	<0.01	−6.03	<0.01	0.042	>0.10
South Africa	9.87	<0.01	−4.78	<0.01	0.069	>0.10
Japan	6.51	<0.01	−3.94	<0.01	0.103	>0.10

**Table 4:** Maximal Lyapunov exponent estimates for CDS log-changes in selected countries.

Country	US	DE	IT	CN	BR	TR	ZA	JP
${\hat{\lambda }}_{\max}$	0.041	0.038	0.046	0.032	0.053	0.061	0.048	0.027
*p*-value	<0.01	<0.01	<0.01	<0.05	<0.01	<0.01	<0.01	<0.05

**Table 5:** Hyperparameter settings.

Component	Parameter	Value
Transfer entropy	Rolling window *W*	250 trading days
	Embedding lags (k,l)	(3,3)
	Neighbour count *K*	5
	Surrogate count *S*	200
	Significance threshold	95th surrogate percentile
Hawkes process	Kernel type	Exponential
	Extreme event threshold α	0.95 quantile
	Estimation method	EM algorithm
	Stationarity check	ρ(Λ)<1
	Sparsification	LR test at 5% level
Composite network	Fusion weight ω	0.55 mean (expanding-window CV)
	Normalisation	Row-wise max scaling
SEI	$\left(\gamma_{1},\gamma_{2},\gamma_{3}\right)$	(0.4,0.35,0.25)

**Table 6:** Top-10 directional transfer entropy edges by sub-period (effective TE in nats).

Pre-COVID-19			COVID-19			Post-COVID-19		
Rank	Edge	TE	Rank	Edge	TE	Rank	Edge	TE
1	US→IT	0.142	1	US→BR	0.287	1	DE→IT	0.231
2	US→ES	0.131	2	US→IT	0.264	2	IT→ES	0.218
3	UK→IT	0.118	3	BR→CO	0.241	3	US→TR	0.197
4	US→BR	0.107	4	IT→ES	0.228	4	UK→DE	0.186
5	DE→IT	0.098	5	CN→KR	0.217	5	DE→FR	0.174
6	CN→KR	0.091	6	UK→ZA	0.203	6	TR→EG	0.168
7	JP→AU	0.084	7	DE→FR	0.194	7	US→BR	0.162
8	BR→MX	0.078	8	TR→RU	0.186	8	RU→TR	0.155
9	IT→ES	0.073	9	US→MX	0.178	9	SA→EG	0.148
10	US→MX	0.067	10	JP→AU	0.171	10	CN→ID	0.141

**Table 7:** Selected Hawkes excitation parameters, ${\hat{\alpha }}_{ij}$, and branching ratios (full sample).

From∖To	US	DE	IT	CN	BR	TR
Excitation coefficients ${\hat{\alpha }}_{ij}$						
US	—	0.087	0.142	0.051	0.168	0.094
DE	0.063	—	0.214	0.032	0.047	0.078
IT	0.048	0.178	—	0.021	0.058	0.103
CN	0.037	0.024	0.019	—	0.042	0.031
BR	0.091	0.036	0.052	0.028	—	0.046
TR	0.054	0.067	0.089	0.018	0.041	—
Branching ratio ρ(Λ)						
Pre-COVID-19: 0.62 COVID-19: 0.84 Post-COVID-19: 0.71						

**Table 8:** Contagion intensity during stress episodes: composite framework versus single-layer baselines.

Episode	TE Only	Hawkes Only	Composite	Amplification (%)
COVID-19 shock (March 2020)	0.214	0.187	0.338	57.9
2022 energy crisis (October 2022)	0.178	0.164	0.241	35.4
2023 banking stress (March 2023)	0.156	0.138	0.217	39.1
Full sample mean	0.094	0.082	0.121	28.7

**Table 9:** Early warning lead times (trading days) before peak drawdowns.

Indicator	COVID-19	Energy Crisis	Banking Stress	Mean
${\mathrm{CI}}_{t}$ (composite)	8	6	5	6.3
$H_{vN}\left(t\right)$	12	9	7	9.3
${\mathrm{SEI}}_{t}$	11	10	8	9.7
Diebold–Yilmaz total [1]	3	2	1	2.0
TE-only ${\mathrm{CI}}_{t}$	6	4	3	4.3
Hawkes-only ${\mathrm{CI}}_{t}$	5	5	4	4.7

**Table 10:** Robustness of the contagion intensity index: coefficient of variation across parameter choices.

Parameter Varied	Range	Mean CI	Std CI	CV (%)
Window *W*	150–350	0.119	0.008	6.7
Threshold α	0.90–0.97	0.123	0.011	8.9
Lags (k,l)	(2,2)–(5,5)	0.118	0.007	5.9
Kernel	3 types	0.121	0.006	5.0
Fractional differencing	d∈{0.05,0.10,0.15}	0.117	0.009	7.7

**Table 11:** Early warning lead time (trading days, mean across three episodes) of the von Neumann graph entropy under alternative Tsallis *q*-parameters.

*q*	0.5	0.8	1.0 (Shannon)	1.5	2.0
Mean lead time (days)	8.7	9.1	9.3	8.9	8.4
Rank corr. with q=1	0.93	0.97	1.00	0.96	0.91

## Data Availability

The sovereign CDS spread data used in this study were sourced from the Bloomberg Terminal (https://www.bloombergchina.com/solution/data-content/market-data/ (accessed on 31 July 2026)) and S&P Global Market Intelligence (formerly IHS Markit; https://www.spglobal.com/marketintelligence/en/ (accessed on 31 July 2026)) under their respective licence agreements. The code supporting this study is available from the corresponding author upon reasonable request.
